# Occult Gastrointestinal Hemorrhage From a Meckel’s Adenocarcinoma: A Diagnostic Dilemma

**DOI:** 10.7759/cureus.59685

**Published:** 2024-05-05

**Authors:** Roham Salman Roghani, Arsia Jamali, Adewale Ajumobi

**Affiliations:** 1 Internal Medicine, Eisenhower Health, Rancho Mirage, USA; 2 Gastroenterology, Eisenhower Health, Rancho Mirage, USA; 3 Medicine, University of California Riverside, Riverside, USA

**Keywords:** meckel’s adenocarcinoma, provocative arteriography, gastro intestinal bleeding, hemorrhage, adenocarcinoma

## Abstract

Gastrointestinal bleeding from Meckel's diverticulum can be challenging to diagnose. We present a case of a 78-year-old man with painless hematochezia. Despite undergoing standard investigations, the source of bleeding remained elusive until arteriography localized bleeding from Meckel's diverticulum. Prompt management involved embolization followed by laparoscopic resection. This case underscores the need to consider Meckel’s diverticulum as a source of obscure gastrointestinal bleeding even in the elderly, as well as the need to use non-conventional diagnostic approaches when standard methods fail. The successful management of the case through embolization and laparoscopic resection highlights the crucial role interventional radiologists and surgeons play in the management of Meckel’s diverticulum-related complications.

## Introduction

Lower gastrointestinal bleeding (LGIB) is a frequently encountered clinical phenomenon. Typically, colonoscopy, nuclear medicine scan, and angiography, including CT mesenteric angiography (CTMA), successfully identify the etiology of the bleeding in most cases. However, a subset of patients presents a diagnostic dilemma, as the source of bleeding remains elusive despite employing conventional procedures [1].

We present the case of a 78-year-old man who presented with painless hematochezia. He underwent colonoscopy, upper gastrointestinal endoscopy, and computed tomography (CT) angiogram, all of which yielded inconclusive results. A nuclear medicine red blood cell scan revealed bleeding activity in the right colon, and a repeated colonoscopy with terminal ileum intubation was unremarkable. Subsequent provocative arteriography, performed with heparin and alteplase, raised suspicion of Meckel’s diverticulum due to active bleeding from the ileal branch of the superior mesenteric artery, initially thought to be the vitelline artery. This was managed with embolization followed by laparoscopic resection.

## Case presentation

A 78-year-old man with a medical history of non-anticoagulated paroxysmal atrial fibrillation presented with painless hematochezia for one day. He did not report previous episodes of gastrointestinal (GI) bleeding. On presentation, he was hemodynamically stable with hemoglobin at 12.8 g/dL. The coagulation profile and serum chemistries were within normal limits.

**Figure 1:**
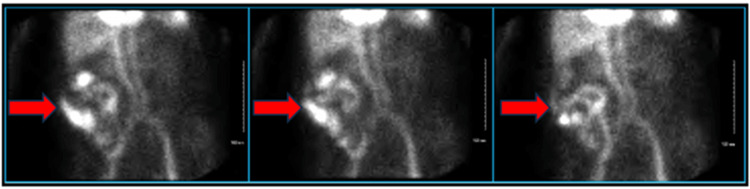
Images of nuclear medicine nuclear red blood cell scan showing bleeding in the right upper quadrant, suspicious for bleeding in the terminal ileum or right hemicolon (red arrows).

**Figure 2:**
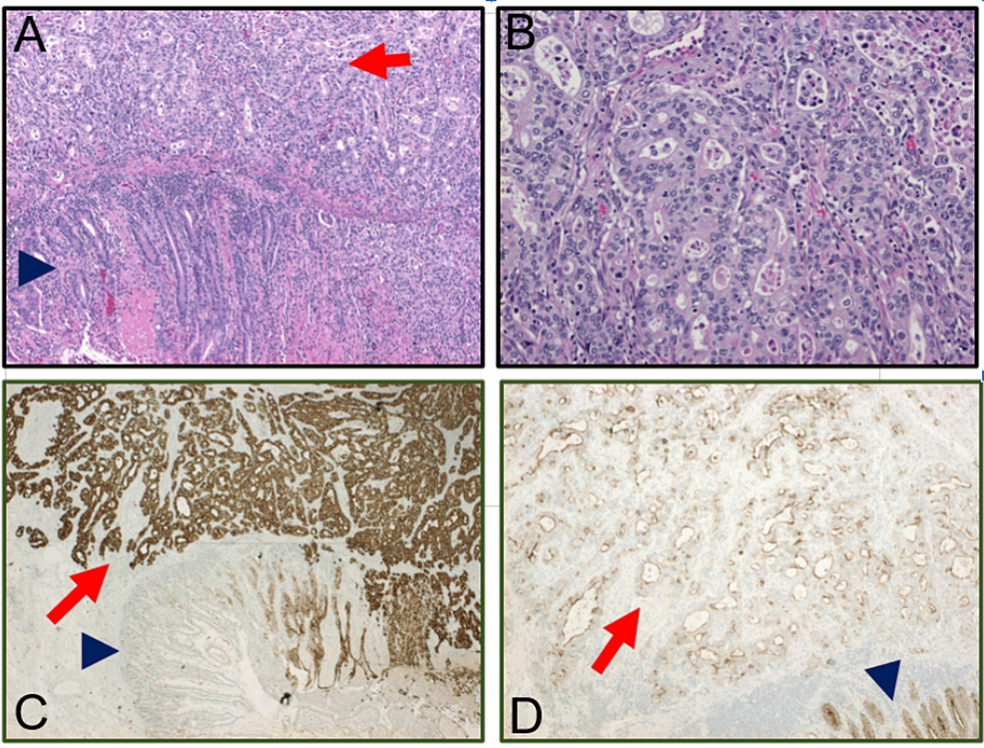
Pathologic assessment of the resected tissues (A) Hematoxylin and eosin staining at 100x magnification showed moderately differentiated adenocarcinoma (red arrow) adjacent to benign small intestinal mucosa (blue arrowhead). (B) High-resolution images with hematoxylin and eosin staining at 400x magnification demonstrated marked architectural distortion, variations in nuclear size and shape, hyperchromasia, and increased mitoses. (C) Immunohistochemistry staining for CK7 at 100x magnification indicated strong staining in the adenocarcinoma (red arrow) and weak staining of the adjacent benign small intestinal mucosa (blue arrowhead). (D) Immunohistochemistry staining for Villin at 400x magnification showing loss of Villin immunostaining in the moderately differentiated adenocarcinoma (red arrow) and normal small intestinal mucosa with strong luminal and membrane staining (blue arrowhead).

A colonoscopy showed the presence of hematin in the terminal ileum and throughout the colon. However, the source of the bleeding was not identified. Repeat colonoscopy with terminal ileum intubation the next day was unremarkable. Due to continued hematochezia, he underwent a CT angiogram of the abdomen and pelvis, which did not reveal the source of the bleeding. He then underwent an esophagogastroduodenoscopy (EGD), which also did not reveal a source of bleeding. Subsequently, a nuclear red blood cell bleeding scan indicated bleeding activity suspected to be in the right colon (Figure 1). Based on this result, he underwent a repeat colonoscopy which showed a normal terminal ileum but re-demonstrated hematin throughout the colon without active bleeding. A subsequent arteriography did not reveal active bleeding. The patient was transferred to the intensive care unit (ICU) for provocative arteriography using a hospital-established protocol of heparin and alteplase. He had an episode of hematochezia in the ICU. Direct arteriography after provocation showed active bleeding from the ileal branch of the superior mesenteric artery, which was thought to be the vitelline artery. Thus, bleeding from a Meckel’s diverticulum was suspected. Coil embolization of the bleeding vessel was performed during angiography. The patient ultimately underwent diagnostic laparoscopy with washout and resection of a perforated Meckel’s diverticulum. Pathology showed a moderately differentiated adenocarcinoma with mucosal ulceration, necrosis, and involvement of all layers of the bowel (Figure 2). Postoperatively, the patient experienced no further episodes of bleeding.

## Discussion

Although the majority of GI bleedings are identified and subsequently managed by upper GI endoscopy or colonoscopy, obscure GI bleeding can be challenging to manage [1,2]. In this case, the source of GI bleeding was not identifiable by colonoscopy with terminal ileum intubation, EGD, CT angiography, direct arteriography, and nuclear medicine red blood cell scan. Faced with ongoing bleeding and diagnostic uncertainty, we proceeded with provocative angiography in accordance with our institutional protocol. This procedure was conducted in the ICU with the simultaneous presence of a general surgeon and interventional radiologist, ensuring a swift response to any potential complications. Capsule endoscopy could have been considered as a diagnostic option, but it was not available at the hospital units at the time of the patient encounter. 

Meckel’s diverticulum is a rare cause of GI bleeding [3], with Meckel’s adenocarcinoma being even rarer [4]. Meckel’s diverticulum is the most common congenital malformation of the upper gastrointestinal tract [3]. It results from an incomplete obliteration of the fetal vitelline duct during the first 8 weeks of gestation. As a true diverticulum, it is lined by typical ileal mucosa. However, most Meckel’s diverticula contain heterotopic tissue, with ectopic gastric mucosa being the most common heterotopic tissue. 

Although Meckel’s diverticula can remain asymptomatic, complications may occur due to the acid secretion by the heterotopic gastric mucosa, leading to ulceration of the nearby ileal mucosa [3]. Although the incidence of Meckel’s diverticulum is equal in both males and females, the frequency of complications is 3-4 times higher in males [5]. While typically, Meckel’s diverticula present in childhood, they may remain undiagnosed until adult life [3,6]. Due to its rarity in adults, it is often missed or its diagnosis is delayed preoperatively [6]. The most common complication in adults is intestinal obstruction with intussusception, with Meckel’s diverticulum serving as the lead point [7]. Other complications include ulceration, diverticulitis, and perforation with subsequent fistula formation. Rarely, individuals with Meckel’s diverticulum may develop torsion, volvulus, and tumors, such as carcinoid tumors, carcinoma, sarcoma, or adenocarcinoma [7]. 

Hemorrhage is a common presentation in both children and adults, with children having red or maroon stools and adults having more melenic stools due to slower colonic transit time [7]. Imaging modalities, such as ultrasound, CT scan, angiography, and MRI, are available to aid in diagnosis; however, the sensitivity and specificity are low [8]. Additionally, the 99m Tc-pertechnetate Meckel’s scan is helpful in diagnosing Meckel’s diverticulum as it could detect ectopic gastric mucosa [9]. Meckel's scan was not performed in our case due to a lack of clinical suspicion for Meckel's diverticulum. Given the patient's age and presentation with painless hematochezia, other etiologies such as diverticulosis, vascular malformations, or malignancies were considered initially. Instead, we opted for other standard diagnostic modalities guided by the patient's clinical presentation and established investigative practices for evaluating gastrointestinal bleeding in adults. We became suspicious of Meckel's diverticulum based on the arteriography, which showed active bleeding from the ileal branch of the superior mesenteric artery, initially thought to be the vitelline artery. Subsequently, the patient underwent embolization and surgical resection of the meckel’s diverticulum. 

There are several teaching points involved in this case. Meckel’s diverticulum can be seen in elderly patients. It can be missed despite intubation of the terminal ileum during colonoscopy. Interpretation of a positive nuclear medicine bleeding scan requires care and attention. The location of activity shows where blood is found at the time of the scan and not necessarily the bleeding site. A provocative arteriography may be required to identify and treat the source of obscure gastrointestinal bleeding. While asymptomatic Meckel's diverticulum may not necessitate surgery, surgical resection becomes imperative for symptomatic cases, particularly those involving bleeding, as there is a potential risk of harboring malignancy.

## Conclusions

In summary, this case underscores the diagnostic challenges of obscure gastrointestinal bleeding, particularly in elderly patients with uncommon conditions like Meckel's diverticulum. Despite a thorough evaluation involving various imaging modalities, the source of bleeding remained elusive until provocative arteriography revealed active bleeding from a Meckel's diverticulum, ultimately leading to surgical resection. The case highlights the need for a high index of suspicion, especially in the elderly, and the importance of considering rare etiologies in cases of persistent gastrointestinal bleeding. It also emphasizes the limitations of conventional imaging techniques and the significance of interpreting positive nuclear medicine bleeding scans with caution. The successful resolution of the case through targeted interventions serves as a valuable teaching point for clinicians managing obscure gastrointestinal bleeding.
